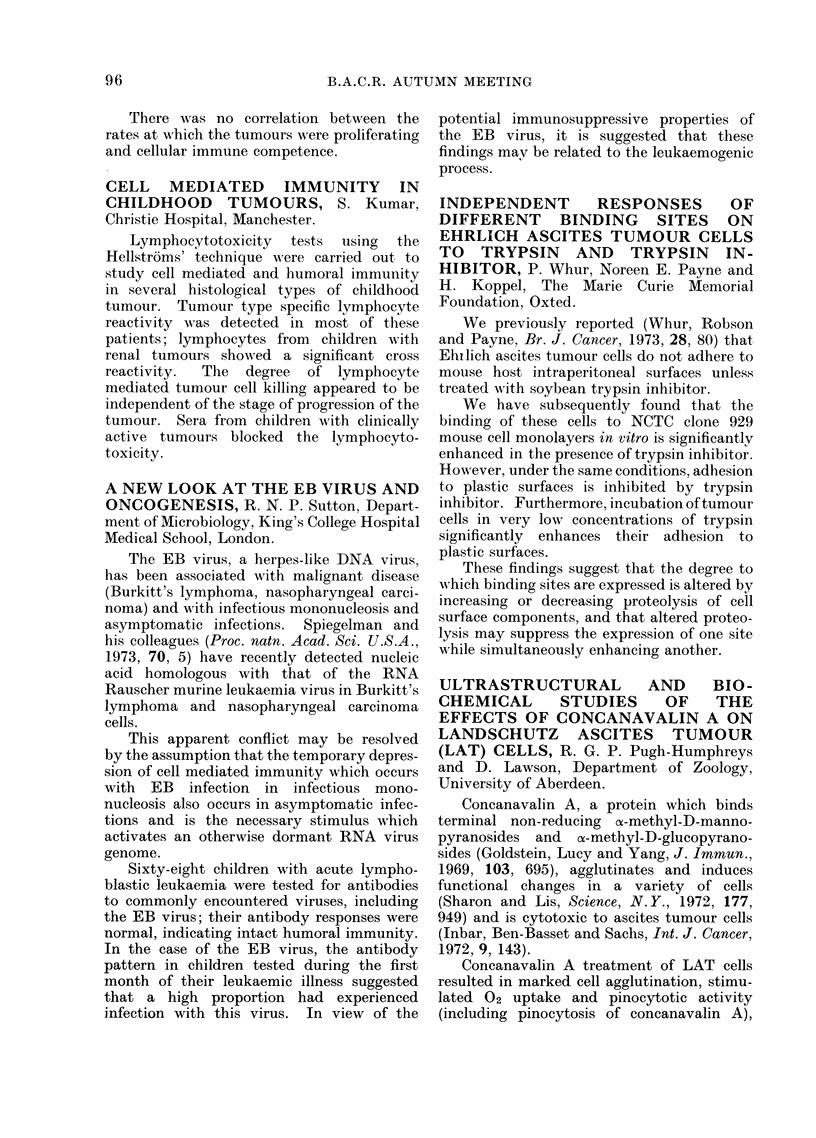# Proceedings: Cell mediated immunity in childhood tumours.

**DOI:** 10.1038/bjc.1974.26

**Published:** 1974-01

**Authors:** S. Kumar


**CELL MEDIATED IMMUNITY IN CHILDHOOD TUMOURS,** S. Kumar, Christie Hospital, Manchester.

Lymphocytotoxicity tests using the Hellströms' technique were carried out to study cell mediated and humoral immunity in several histological types of childhood tumour. Tumour type specific lymphocyte reactivity was detected in most of these patients; lymphocytes from children with renal tumours showed a significant cross reactivity. The degree of lymphocyte mediated tumour cell killing appeared to be independent of the stage of progression of the tumour. Sera from children with clinically active tumours blocked the lymphocytotoxicity.